# Exploring Determinants of Patient Adherence to a Portal-Supported Oncology Rehabilitation Program: Interview and Data Log Analyses

**DOI:** 10.2196/rehab.6294

**Published:** 2017-12-14

**Authors:** Hendrik P Buimer, Monique Tabak, Lex van Velsen, Thea van der Geest, Hermie Hermens

**Affiliations:** ^1^ Department of Biomedical Signals & Systems Faculty of Electrical Engineering, Mathematics and Computer Science University of Twente Enschede Netherlands; ^2^ Department of Biophysics Faculty of Science Radboud University Nijmegen Netherlands; ^3^ Telemedicine Group Roessingh Research & Development Enschede Netherlands; ^4^ Research Center IT + Media HAN University of Applied Sciences Arnhem Netherlands

**Keywords:** telemedicine, rehabilitation, patient portals, treatment adherence, compliance

## Abstract

**Background:**

Telemedicine applications often do not live up to their expectations and often fail once they have reached the operational phase.

**Objective:**

The objective of this study was to explore the determinants of patient adherence to a blended care rehabilitation program, which includes a Web portal, from a patient’s perspective.

**Methods:**

Patients were enrolled in a 12-week oncology rehabilitation treatment supported by a Web portal that was developed in cooperation with patients and care professionals. Semistructured interviews were used to analyze thought processes and behavior concerning patient adherence and portal use. Interviews were conducted with patients close to the start and the end of the treatment. Besides, usage data from the portal were analyzed to gain insights into actual usage of the portal.

**Results:**

A total of 12 patients participated in the first interview, whereas 10 participated in the second round of interviews. Furthermore, portal usage of 31 patients was monitored. On average, 11 persons used the portal each week, with a maximum of 20 in the seventh week and a drop toward just one person in the weeks in the follow-up period of the treatment. From the interviews, it was derived that patients’ behavior in the treatment and use of the portal was primarily determined by extrinsic motivation cues (eg, stimulation by care professionals and patient group), perceived severity of the disease (eg, physical and mental condition), perceived ease of use (eg, accessibility of the portal and the ease with which information is found), and perceived usefulness (eg, fit with the treatment).

**Conclusions:**

The results emphasized the impact that care professionals and fellow patients have on patient adherence and portal usage. For this reason, the success of blended care telemedicine interventions seems highly dependent on the willingness of care professionals to include the technology in their treatment and stimulate usage among patients.

## Introduction

Over the last couple of years, the use of telemedicine applications within health care has increased. The term telemedicine refers to health services that enable patients to receive treatment in their daily living environment, whereby distance between health care professionals and patients is bridged by information and communications technologies (ICTs) [1]. Therefore, telemedicine can be used as a stand-alone treatment, or it can be combined with face-to-face treatments to form so called blended care treatments [2]. Telemedicine is believed to provide opportunities to increase the efficiency and effectiveness of health care services, resulting in improved health outcomes [3,4]. However, telemedicine applications often do not live up to these expectations and often fail once they have reached the operational phase [5,6]. Especially in cases where users need to use telemedicine over time, declined usage is prevalent [1,5]. Why is it so difficult to successfully implement telemedicine applications in health care treatments and to avoid nonusage over time? And why is it so hard to make patients use technologies—that are designed to improve their treatment outcomes over time?

One of the possible explanations could be found within the concept of patient adherence, which is the extent to which the patient’s behavior matches the agreed recommendations of the prescriber. As with more traditional treatments, telemedicine requires patients to be active users over time to be successful and have a chance of positive clinical outcomes [3,7,8]. In traditional health care, patient adherence is known to be an important factor when it comes to the success of health care treatments and medication intake [9]. Low patient adherence is known to lead to increased health care costs and negative health outcomes [8,10]. As far as we know, there has not been a lot of research exploring the determinants of patient adherence in blended care treatments in which a lot of interaction between off- and online factors are likely to be at play. In this study, the determinants of patient adherence to a blended care rehabilitation program, including a Web portal, were explored from the perspective of both patients and care professionals. This paper focuses on the patient’s perspective.

## Methods

### Context

This study was set around a portal designed for a blended care rehabilitation program aimed at supporting cancer survivors who got out of primary care to cope and live with the consequences of the disease on their life. In an intensive 12-week program, patients were supervised by a multidisciplinary team of care professionals such as social workers, rehabilitation physicians, and physiotherapists. Patients were assigned to groups with fellow patients and participated in a variety of group activities and sessions. Besides group sessions, which took place three times per week at the rehabilitation center, patients were required to do additional individual activities and exercises at home.

These home activities were supported by a telemedicine intervention in the form of an online portal. To make the portal as fitting to the needs of patients and care professionals as possible, the modules in the portal were designed and developed following a user-centered approach in close cooperation with care professionals and patients from the program. Before introduction, the usability of both the patient’s and the care professional’s side of the Web portal was evaluated and improved.

**Figure 1:**
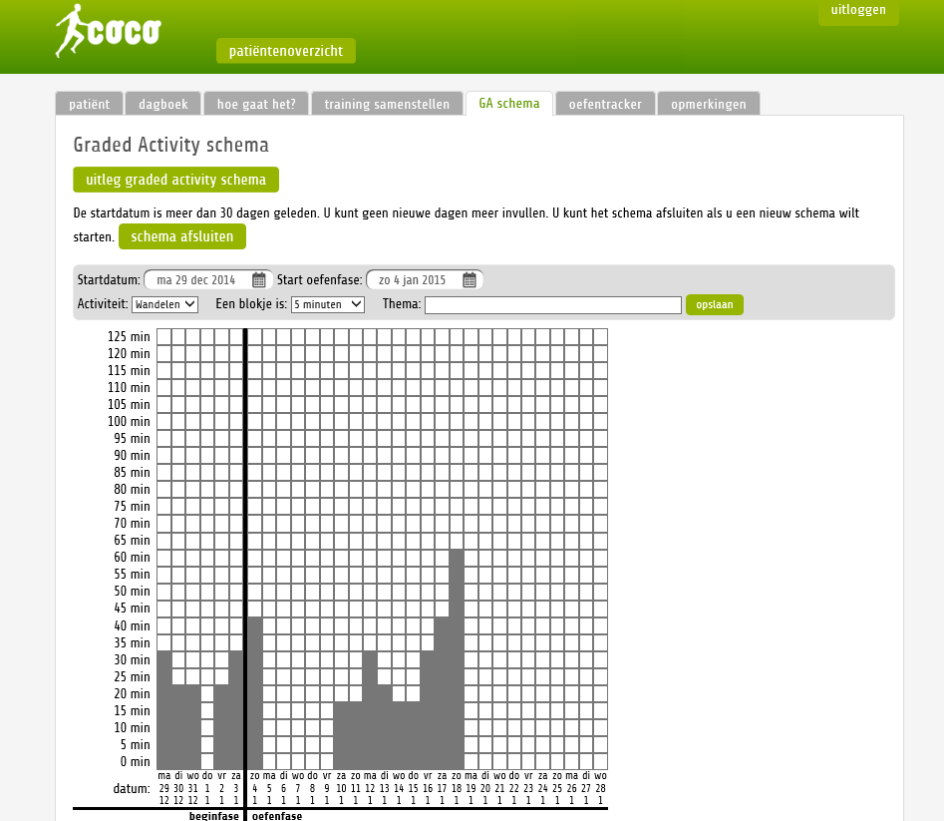
Screenshot of the graded activity scheme (physical self-report diary) on the Web portal subject to this study.

The primary goal of the portal was to support the rehabilitation treatment and to facilitate self-management by patients. Therefore, the portal contained the following modules: (1) information about the program and the disease; (2) activities and exercises, with video instructions about individual exercises to enable patients to do their exercises independently at home; (3) self-report diaries (see Figure 1), which enabled monitoring of physical and mental progress during the program; and (4) a message function, enabling patients to leave messages for care professionals and enabling care professionals to effectively target their care to the needs and wishes of patients during sessions at the rehabilitation center. Usage of the portal was not mandatory, although it was strongly advised to patients to use the information from the portal for home exercises. After the rehabilitation program ended, the Web portal remained available for several months to patients without explicit support by care professionals.

### Participants

There were 2 groups of participants in this study: patients and care professionals. This paper focuses on determinants of patient adherence to the portal from the perspective of patients. All patients enrolled in the oncology program in the period of September 2014 to February 2015 were approached to participate in the study. Participation in the study was voluntary, and an informed consent was obtained before participation. The study was approved by a medical ethical committee. A total of 12 patients agreed to participate in the study; 11 of the participants were females, with an average age of 53.8 (standard deviation [SD] 7.2) years. Furthermore, portal usage data was analyzed of 31 patients, of which 29 were females and 2 males, with an average age of 52.4 (SD 8.5) years, who gave permission to use their data upon log-in to the portal for the first time.

### Procedure

To explore the behavior within the program, 2 rounds of open interviews were conducted among the participating patients and care professionals. A list of topics (see Textboxes 1 and 2) was used to structure the open interviews. The first interview took place within 3 weeks after the start of the program, whereas the second interview was scheduled toward the end of the program. In addition to the interviews, logged usage of the portal was analyzed to determine the actual use of the portal during the 12-week program. Because the portal was available to the patients after the rehabilitation treatment had ended at the center, it was decided to collect usage data over a 10-week follow-up period too.

**The topics addressed in interview 1 conducted with patients.**

- Living with the disease
  - Impact on daily life
- Treatment
  - Goals
  - Expectations
  - Influence on others
- Role of the portal
  - Acceptance of technology
  - Expectations
  - Goals
- Near future
  - Behavioral intention treatment
  - Behavioral intention portal usage
  - Expectations

**The topics addressed in interview 2 conducted with patients.**

- Recap
  - Impact on daily life
  - Treatment
  - Influence of others on treatment
- Role of the portal
  - Portal usage
  - Perceived usefulness
  - Influence on others on portal usage
- Preview on future
  - Self-efficacy
  - Portal usage after treatment

### Instrument

The aim of the first interview with the patients was to gain insights in their behavioral intentions for the treatment, including their use of the portal. According to the theory of planned behavior (TPB) [11,12], behavioral intentions are indications of how much an individual is willing to perform a particular behavior. The second interview focused more on actual behavior and experiences with the rehabilitation program and the portal. To do so, health behavior was explored from the perspective of 3 different human behavior models used to identify determinants of health behavior within the treatment. Additional determinants were derived from literature describing patient adherence in various health care contexts. This was done to ensure a comprehensive overview of behavioral determinants. The determinants derived from the TPB were used to explore the behavioral intention of the patients. The TPB is built on the assumption that all behavior is intentional and is determined by one’s attitudes, normative beliefs, and perceived behavioral control [11,12]. It is known to be applicable to health care contexts. We also explored determinants derived from the health belief model (HBM) to take preventive health behavior topics into account, such as perceived susceptibility, perceived seriousness, perceived benefits and barriers to taking action, and cues to action [13,14]. Patients in this study had received primary care for their disease before, making the latter three factors from the HBM applicable and useful within the context of this study. Third, determinants derived from the technology acceptance model (TAM), such as perceived ease of use and usefulness, were used to explore reasons for usage and nonusage of the portal, which was an essential element of the treatment [15,16]. Finally, various determinants used in this study were derived from the model of supported accountability (SA) [5], which describes how patient adherence is enhanced by human support from care professionals and moderated by a patient’s motivation and the type of communication technology used. This theoretical background resulted in the topic list that can be found in Textboxes 1 and 2, as well as in themes for analyzing the interviewee’s responses (see Table 1).

### Data Analysis

#### Usage Data

Portal usage was determined by counting and analyzing sessions focusing on the individual page visits, which were categorized into one of 3 categories (information, activities and exercises, and self-report diaries). It was not possible to count usage of the message function, as this function was implemented into the various other functionalities, and the number of messages sent back and forth was not available. Furthermore, it was decided to monitor one of the activity self-management tools, being the graded activity scheme, separately. The number of page visits was analyzed to determine how much the 4 categories were used during each week of the program.

#### Interviews

The interviews with patients and care professionals were recorded with a voice recorder. The audio files were transcribed and divided into short episodes. All episodes were coded into different categories following a thematic analysis method [17,18]. The initial code list of determinants was based on the TPB [11,12], HBM [13,14], and TAM [15,14], theoretical models, complemented with possible determinants of health behavior derived from literature that explains possible determinants for patient adherence, such as the model of supportive accountability (SA) [5], which includes (among other determinants) patient’s expectations, motivation, and voluntariness to accept the influence of the care professional [5]; perceived disease severity [13,19]; and patients-to-care professional communication [5,8,13,20-22]. Coding of the episodes was done by 2 independent coders who reached consensus considering the assigned codes after a low agreement at first sight.

## Results

### Usage of the Portal

**Figure 2:**
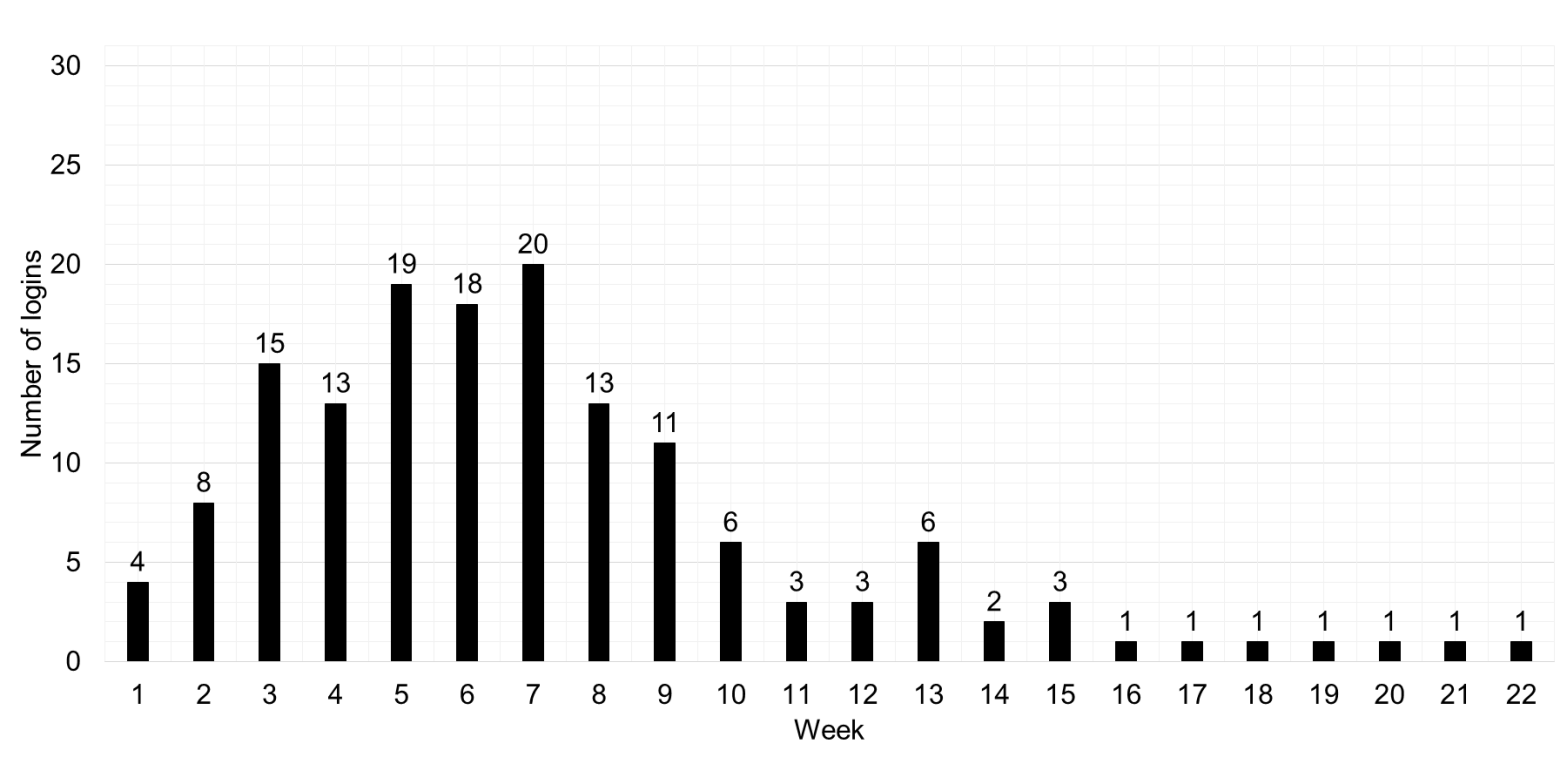
Overview of the number of individual users per rehabilitation treatment week (N=31).

How much was the portal used by the patients? The data were collected from 31 anonymous patients who logged in to the portal at least once, including the respondents of the study; 2 of the interviewees never succeeded in getting into the portal. Figure 2 shows the number of patients who logged in to the portal in each week of the rehabilitation treatment and during a 10-week follow-up period after the treatment. The graph shows that patients used the Web portal more during the treatment phase, whereas after the treatment their usage drops. The number of persons who logged in to the portal increased in the first weeks of the treatment and decreased from week 7.

**Figure 3:**
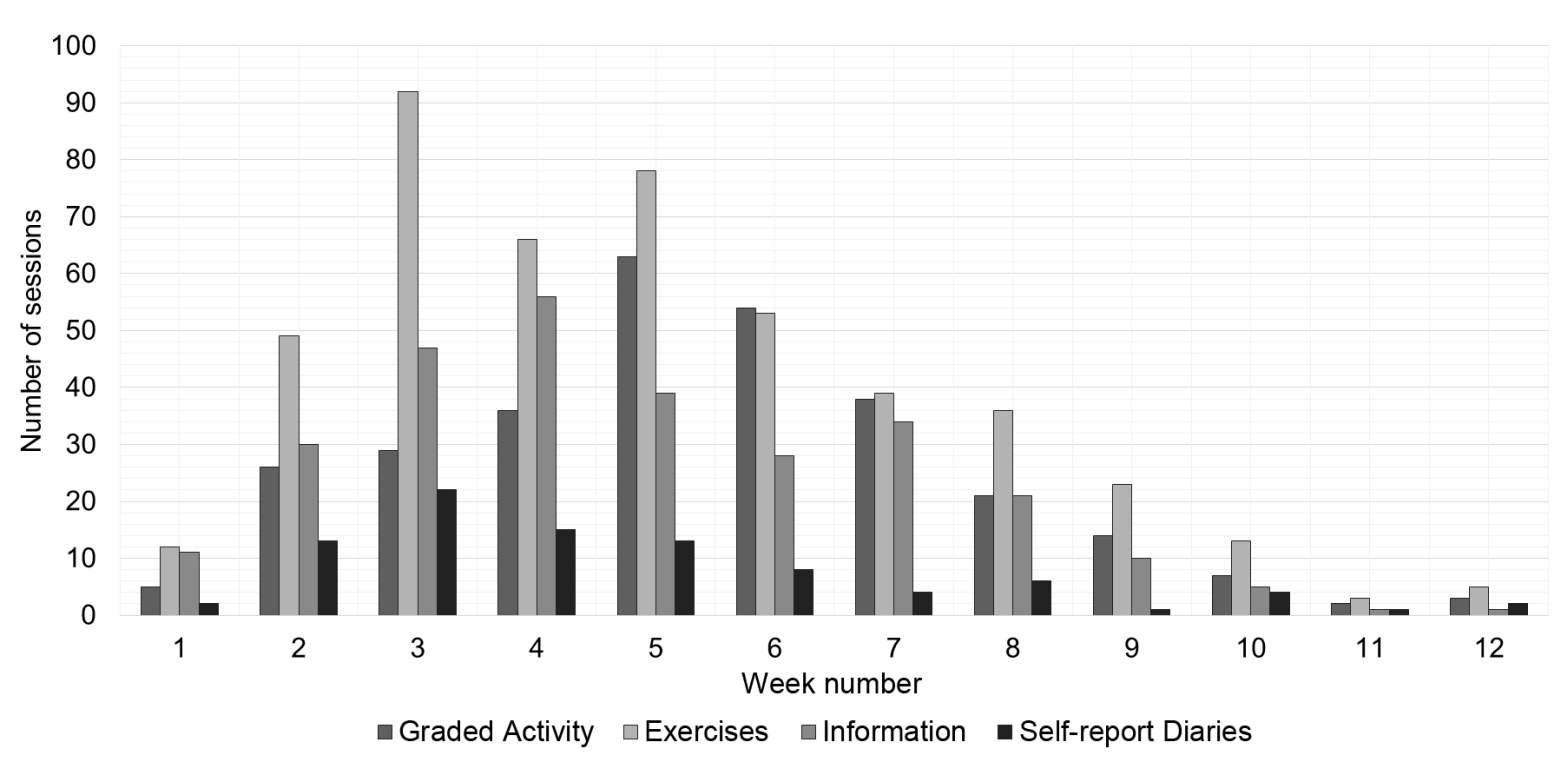
Overview of the number of sessions ordered by functionality.

As mentioned before, the Web portal included 4 functionalities, addressing different tasks within the treatment: graded activity scheme (a physical self-report diary; 21 different individuals), Web-based exercises (30 different individuals), information (30 different individuals), and self-report diaries (20 different individuals). It was not possible to analyze the message function. Figure 3 shows the number of sessions for each functionality per week. The graph shows a similar pattern as Figure 2, with higher use in the first weeks and a decreased use over time in which the graded activity (average of 15 sessions per week) and exercises (average of 22.9 sessions per week) were the most visited functionalities.

Important findings that the usage data showed were the limited use of the portal, declined usage over time, and the fact that almost no one was inclined to use the portal after the treatment had ended (even though it was deliberately kept available to patients after the 12-week program). These findings led us to explore the determinants for usage and nonusage of the portal from the interviews that were conducted among patients.

**Table 1:** Determinants of adherence. Overview of the number of codes assigned to relevant episodes in the interviews. Descending from the most often to the least mentioned themes.

Theme of episode	Derived from	Total codes (N=1010) n (%)
Extrinsic motivation cues	TPB^a^ [11,12]	181 (17.92)
Expected benefits of the treatment	HBM^b^ [13,14]	118 (11.68)
Adherence to the portal^e^	TAM^c^ [15], TPB [11,12]	111 (10.99)
Perceived impact of the disease	HBM [13,14]	95 (9.41)
Perceived ease of use	TAM [15]	87 (8.61)
Perceived usefulness of the portal	TAM [15]	79 (7.82)
Adherence to the treatment^e^	TPB [11,12], HBM [13,14]	64 (6.34)
Intrinsic motivation cues	TPB [11,12]	63 (6.24)
Expectations of the portal	-	44 (4.36)
Expectations of the professional	SA^d^ [5]	40 (3.96)
Situational motivation cues	SA [5]	37 (3.66)
Usefulness of other technologies	TAM [15]	19 (1.88)
Facilitating conditions	TAM [15]	15 (1.49)
Voluntariness	TAM [15], SA [5]	15 (1.49)
Previous experience with technology	TAM [15]	13 (1.29)
Attitude toward technology	TAM [15]	10 (0.99)
Trust in technology	-	10 (0.99)
Perceived responsibility	-	9 (0.89)

^a^TPB: theory of planned behavior.

^b^HBM: health belief model.

^c^TAM: technology acceptance model.

^d^SA: supported accountability.

^e^*Adherence to the treatment* and *adherence to the portal* codes were used to classify whether episodes were related to portal or treatment related behavior.

### Interviews

What exactly were the reasons for the decline in usage that was found? In the interviews, the determinants for behavior and how it reflected within the treatment were explored. Interviews were transcribed and divided into episodes. The 12 patients made 763 episodes that were considered relevant over the 2 interviews conducted. Table 1 shows an overview of the number of episodes made for each theme and how this relates to the total number of episodes.

The determinants that were referred to in more than 50 episodes (5%) are described in detail in the following sections. Reported in order; perceived impact of the disease, expected benefits of the treatment, motivational cues, perceived ease of use and perceived usefulness of the portal, ultimately leading to conclusions about adherence.

#### Perceived Impact of the Disease

What was the impact of the disease of the patients on their daily lives before the treatment and how did this impact change over the course of the treatment? All persons included in the rehabilitation treatment have had some form of cancer, of which the majority had breast cancer. During the first interview, 44 episodes where coded as disease, 13 of which were negative, and 3 were positive. Seven respondents stated that the biggest impact of the disease, after receiving primary care treatment, was the fact that they were tired all day, both mentally and physically:

It [the disease] has a lot of influence, as I am tired the entire day. With everything I do, I am easily tired, which is frustrating.

48, female

After the 12-week program, the majority reported to have made progression in the way they perceive the impact of their disease. Most of the health problems discussed in the first interviews were also mentioned in the second interview, although respondents perceived they had improved their ability to accept their changed self and perceived an improved physical condition. On the other side, there were some respondents who experienced a decline in their health, which limited their ability to fully engage in the treatment. A 68-year-old male respondent explained that he was planning to use the portal, together with others, after the treatment at the center had ended because he felt that he had much more physical progress to make before he would be satisfied. On the other hand, a 57-year-old female respondent stated that she had not used the portal over the last week because she was ill and had some issues with herself, and a 58-year-old female patient emphasized that the portal “...should to be supportive and not a challenge, because we have enough challenges already while we are here.” Therefore, the perceived impact of the disease that influenced both the physical and mental condition of the patients seemed to have an influence on patient adherence both in the off- and online treatment.

#### Expected Benefits From the Treatment

The second possible determinant that was discussed with the patients concerned their goals and expectations from the treatment. When asked about the expectations about the treatment, all respondents responded positively. Every patient expected to be able to achieve the goals that they had set before initiating the treatment. In total, 70 episodes were coded into this theme, of which 29 were positive, and 5 were negative. The most important goals that patients had set for themselves considered improvement of physical condition, mental health, and acceptation of themselves after going through an impactful disease:

Not desperately trying to get back to how it used to be, because that is not...But just getting used to the new setting, new situation, with my new body, my new head. I have to find my place and feel comfortable again.

47, female

Respondents were confident that with the help of the care professionals and their fellow patients, they would be able to achieve those goals.

By the end of the rehabilitation program, the respondents remained very positive about the treatment and largely believed their expectations were met (48 episodes, 3 negative vs 26 positive). Respondents were particularly positive about the multidisciplinary approach of the treatment team, physiotherapists, social workers, and psychologists:

I have gained a lot from sports. And the wise lessons from social workers, [...], the conversations with each other and the psychologists. The combination of everything was really good for me.

58, female

Furthermore, the fact that the treatment was offered in groups made it a lot easier for patients to cope with the treatment and the challenges alongside it

The main strength of the treatment was the group. If I had to do it on my own, then I would not have made so much progress.

47, female

Finally, and most importantly, all respondents felt that they had achieved, and in some cases even surpassed, their expectations. The most often mentioned achievements are increased awareness on how to cope with the disease and an increased physical condition:

I have learned to cope better with it. I did learn that you should not always attempt to do everything at once, but that you spread tasks over the entire day.

48, female

None of the respondents mentioned the portal used in the intervention as a positive feature of the treatment, which was no surprise considering the quick decline of use of the portal.

#### Extrinsic Motivation Cues

What was the influence of others on the behavior of patients within the treatment? Extrinsic motivation cues were most often discussed in both interviews, and based on the responses, it is fair to conclude that others had an influence on the behavior of the respondents within the rehabilitation treatment. Seven respondents were referred to, or enrolled in, the treatment by their primary care providers. After being asked about the influence of being enrolled for the treatment, a 58-year-old female respondent stated that this “…makes it more special, which makes you think, I am going to do it.”

Respondents expected that during the treatment, both their fellow patients as well as care professionals would be able to influence their behavior:

Yes, I really feel like I belong here, being in a group of 8 who all have the same disease. And that there is a lot of experience, that everyone knows...The social worker, motor therapist, they know how it works, and what is on your mind and what is tough

47, female

Respondents had the intention to follow instructions provided by the care professionals, and some were expecting such instructions to occur:

But I am not like I do that [look at the portal] every day. I really need someone to tell me to do some exercises. In that case I have a driving force that makes me do so.

57, female

Furthermore, the treatment group, existent of patients who have a shared medical background, was often mentioned as a very positive feature of the treatment. Especially the fact that patients have common understanding of the impact that the disease had on their lives was perceived as a positive thing:

Being together with others who suffer the same disease, although in different ways, but...cancer is cancer. You learn to talk about it. It is easier with fellow patients than with outsiders, or your partner. In such cases, you have the feeling they have heard it all.

48, female

During the second round of interviews, however, an example arose of the negative influence a strong group feeling might have on patient adherence and in this case, usage of the portal. Two patients were unable to log in to the portal, which influenced both care professionals and patients associated to their treatment group:

We collectively quit using the portal, because 2 persons could not join in. Two persons who cannot login on 7 participants is a lot. It drove them crazy, as they reported the issues and felt nothing was done about it. [...] And then we decided together to drop it!

58, female

One respondent even stated that the portal became a topic that was avoided during group sessions because there were some patients who got annoyed by the fact that there were some who could not use the portal at all. Thus, besides the direct impact on the 2 persons that could not use the portal, this inability to use the portal resulted in a lower willingness to use the portal across their entire treatment group. Luckily, there were also some positive signals. A 46-year-old female respondent reported that “if someone told me they had watched a functionality, I would check it at home too.” And she was not alone in this. Others also stated they would have used the portal more if usage would have been stimulated more by the care professionals. The findings from these interviews really emphasize the impact that both care professionals and fellow patients have on the willingness to use the portal within the treatment. Especially, stimulation by care professionals and a positive attitude within the group toward the portal seem to be positive determinants for patient adherence.

#### Intrinsic Motivation Cues

According to the literature, patients who are more intrinsically motivated required less stimulation [5]. So how was the intrinsic motivation of the patients included in this study? A total of 36 episodes across 11 interviews were assigned to intrinsic motivation cues. The general intrinsic motivation of the respondents was positive (18 positive vs 6 negative episodes in the first round of interviews). Respondents were highly motivated to actively participate within the treatment to get back into full participation within the society:

I think this treatment is so beautifully set up, this is a chance that I get to get my life back on track. Of course, I will seize to grab this opportunity with both hands.

47, female

One person even stated he had already put effort into training to strengthen his physical condition before signing up for the treatment, which is proof of his strong intrinsic motivation to get better. In line with the first round of interviews, participants within the treatment remained intrinsically motivated during their entire treatment (63 episodes, 5 negative vs 12 positive):

I was full engaged, and still am. [...] I you want to achieve something, you will give your 100%.

60, female

However, as a 47-year-old female respondent stated, it did vary how people translated this motivation into action:

I believe everyone was seriously engaged within the treatment. Though I was a bit surprised that some participants took a week or a weekend off and missed treatment days because of it. I decided that during these 12 weeks all appointments associated with the treatment received highest priority. [...] I felt it was a now or never kind of story.

The responses to the questions concerning intrinsic motivation were quite uniform, in the sense that everyone seemed to be intrinsically motivated to engage in the treatment. Although this might be a very strong determinant for their willingness to engage in group- and other offline sessions, it is unlikely that this was an important determinant for usage of the portal.

#### Perceived Ease of Use of the Portal

Could it be that the determinants for the usage patterns that were found are originating from the TAM [15]? First, the responses considering the ease of use of the portal were mixed. Over a total of 49 coded episodes, 12 were negative and 11 were positive. Positive remarks were made about the easiness with which the contents of the portal could be found and used, whereas negative remarks primarily focused on difficulties accessing the portal. First, there were 2 respondents who reported difficulties to log in to the portal. The second, and more prevalent problem, was the fact that the portal was only designed for usage from a laptop or desktop computer. This was experienced as a barrier to use the portal by 5 respondents, who explicitly stated they preferred using such a portal from a tablet:

I prefer to use it on the tablet. Some parts do work, while others do not. Those parts do not fit on the screen properly. […] A tablet is a bit easier to pick up.

47, female

During the second round of interviews, responses considering the ease of use of the portal were mixed. Out of 87 quotes, 15 were negative and 11 were positive. As mentioned before, 2 persons dropped out of the treatment as they were unable to log in to the portal and felt insufficiently supported to resolve those issues. On the other hand, many respondents who could log in described the portal as easy, simple, and “easier than the information folder,” 48, female. The primary critique on the portal remained the inaccessibility of the portal on devices other than laptops and computers:

I would like to be able to use the portal on my tablet. It is a shame that I have to grab the laptop to use it, which makes it less convenient to use it.

47, female

Another important comment, stated by 2 respondents, is the fact that users of the portal were unable to see how the content presented on the portal was related to specific parts of the treatment. In other words, it is important that the content on the portal has a strong link to the treatment:

All the content is present, but you are forced to find it yourself. [...] I do not take the time to figure everything out, because I am mentally unable to do so.

49, female

Finally, it is important to understand that the patients who were included had faced a lot of mental and physical challenges and were not willing to accept a telemedicine intervention as another challenge within their treatments:

I only go for the easy way now, if I am honest. I do not take time to figure everything out, because I am not up for it now.

49, female

The interviews with patients showed that the perceived ease of use is very likely to determine the willingness of patients to use a portal in the treatment, especially if the portal is perceived as difficult to use. This emphasized the need for a portal that is easy to access from different devices, provides information that is easy to find and to understand, and is backed up by sufficient facilitating conditions to resolve any issues that patients encounter while using it.

#### Perceived Usefulness of the Portal

During the first interviews, some patients already had some brief experience with the portal, and mostly positive comments were made about the usefulness of the portal with 19 positive and 4 negative quotes. However, there were already 2 respondents who did not feel the need for the portal within the treatment. The other respondents were positive about the usefulness of the portal and its functionalities. Some important reasons for a positive perceived usefulness were the fact that the portal can be used as reference material for exercises and information, a way to monitor progress, a way to communicate with care professionals (although there was a person who wished for more communication abilities), and to find treatment schedules:

It supports the activities that we do here at social work, occupational therapy, and group sessions. It enables you to watch instructions at home. We also have a folder with information, which I do read, but the video instructions on the portal are much easier.

47, female

During the second interviews, respondents were largely positive about the usefulness of the portal (46 codes, 5 negative vs 21 positive). Functionalities that were perceived as useful were the message function (“It was fun to see that these notes were read by care professionals and that they responded or asked questions about the remarks,” 47, female), the graded activity scheme, and the video instructions (“I use the portal daily, especially the mindfulness exercises,” 49, female). A 47-year-old female respondent was overwhelmed by the amount of available information and advised to “...make content on the portal available in doses,” which might help to guide users of the portal to the content that is required in a particular week and increase the earlier discussed fit with the treatment. Furthermore, the blended care caused for some confusion. Because information was both available in traditional information folders and the portal, some respondents were confused as to what was expected from them:

It has to be either the portal or the information folder.

48, female

Several respondents, including a 47-year-old female respondent, emphasized that they intended to visit the portal on a regular basis after the treatment had finished to print and save information that she thinks could be useful in the future. Another reason to visit the portal after the treatment was to see the video exercises that could be found on the portal. However, the fact that on average only 1 patient per week visited the portal makes it unlikely that all patients who said so did. It can be concluded that the portal was perceived as useful by the participants, although that this was not the main determinant to use it or not.

#### Adherence to the Overall Treatment

How do the previously mentioned determinants influence actual adherence within the treatment? The overall adherence to the treatment seemed to be sufficient. Patients were eager to participate in the offline part of the treatment, of which they had very positive expectations beforehand. After the treatment, patients reported positive experiences about the treatment plan that was followed while visiting the rehabilitation center. Intrinsic motivation (getting life back on track), the perceived impact of the disease (both physically and mentally), expected benefits from the treatment (multidisciplinary results and care professionals), and the intensity of the contact with fellow patients were reasons to stay adherent to the treatment.

#### Adherence to the Portal

On the contrary, the analyzed portal usage of patients was rather low. How is it possible that if patients were intrinsically motivated and had positive expectations about the treatment in general, their interest in using the portal was generally low? The 4 most important determinants of portal usage found in this study were (perceived) impact of the disease (being physically or mentally unable to use the portal), extrinsic motivation cues (strong or poor stimulation from care professionals and fellow patients to use the portal), perceived ease of use (especially the ease with which the portal could be visited), and perceived usefulness (influenced by the fit of the portal its content with the general treatment program and communication about usefulness and application from care professional to patient).

## Discussion

### Principal Findings

The main objective of this study was to explore determinants for patient adherence to portal-supported rehabilitation treatments from the perspective of both patients and care professionals, where this paper focused on the patients’ perspective. When it comes to the offline part of the treatment, patients were generally positive and willing to engage in the treatment. All respondents were intrinsically motivated to get their lives back on track after suffering from a life-threatening disease and wanted to fully participate in society again. Participants were very positive about the multidisciplinary approach of the treatment, had positive expectations about the treatment, intended to fully engage to the program, and had good hopes to get their lives back on track with the support of care professionals.

At the start of the treatment, patients had the intention to use the portal offered alongside it. However, when it comes to portal usage, adherence seemed to be much lower. Whereas an increase in portal usage was seen over the first weeks of the rehabilitation program, it quickly declined after the seventh week of the treatment. After the treatment at the rehabilitation center had ended, on average, only 1 of 31 patients visited the portal.

Even though the portal was designed and developed in close cooperation with care professionals and patients and patients seemed to be generally motivated to stay adherent to the treatment, the usage of the portal remained low. How can this be explained? From the interviews, it appeared that 4 determinants were particularly influencing adherence to the portal: perceived impact of the disease, extrinsic motivation cues, perceived ease of use, and perceived usefulness. Although the 4 determinants were described separately earlier in the manuscript, analyses learned that they were strongly intertwined.

First and foremost, the study showed how important it is for a portal to be truly embedded into the overall program design for it to be used by patients in the treatment. We would like to emphasize the role that care professionals play, as extrinsic motivators, when it comes to increasing awareness among patients about the importance and usefulness of the portal. This is in line with earlier studies that emphasized the role of the care professionals and human support as a strong motivator for health behavior [5,8,21]. The behavior of care professionals and their off- and online communication with patients was reported by patients as a strong incentive to use the portal. In the few weeks that usage of functionalities on the portal was stimulated, portal usage increased. This finding emphasizes the crucial role that care professionals play in the potential success of portal applications in rehabilitation programs. Clear communication from care professionals to patients about the usefulness of, and benefits derived from the portal, by emphasizing a strong link with the offline program, might increase awareness of the usefulness of the portal and stimulate active usage of a portal in the treatment [19,20]. These observations strengthen the suggestion that a telemedicine intervention will not be successful unless care professionals are to increase the awareness among patients about the importance and usefulness of the ICT within the treatment.

A second group that influenced behavior in the treatment and willingness to use the portal were the fellow patients. Because there was such a strong focus on the group during the treatment, the attitude held within the group toward group activities and usage of the portal influenced the intended behavior. Patients could stimulate each other to engage in group sessions and inform each other about information to be found on the portal. On the contrary, the group also showed negative influence on the behavior of patients, which was showed in the case where 2 persons of a group could not use the portal. They felt insufficiently supported to resolve these issues, leading to a bad attitude toward the portal within the group, which ultimately led to a group decision to quit using the portal.

Finally, the perceived impact of the disease, which was derived from the HBM [13,14], mostly seemed to be a barrier toward portal usage. Patients indicated that the severity of their disease influenced their ability to engage in the treatment and their willingness to use the portal. Some explained that they were physically and mentally unable to use the portal. Patients explicitly stated they did not want to commit to a portal that was perceived as an additional burden to their already intensive rehabilitation program, which is in line with findings of earlier research [19]. This emphasized the need for a portal that is easy to access and use. Where earlier studies found disease severity to be a determinant for general health behavior [13,19], this study showed it is also likely to determine willingness to use a portal supportive to the treatment.

### Strengths and Limitations

What are the strengths and weaknesses of our study? The study was conducted with a low number of participants because the influx in the rehabilitation program was low. Furthermore, many of the approached patients perceived participation in the study as an extra burden to the treatment, and they did not want to commit their precious time to it. The portal did not fully fit the needs of the patients and care professionals involved in the rehabilitation treatment. This resulted in the fact that care professionals were not always willing to apply the portal in the treatment. This made them unsuitable candidates for stimulating usage of the Web portal among patients. Finally, 2 patients that enrolled in the treatment encountered issues with the portal and felt unsupported when trying to address and resolve these issues. Furthermore, the portal was offered rather independent from the treatment and appeared to be insufficiently incorporated within the treatment. It would be interesting to investigate the determinants once these problems are taken care of.

### Conclusions

In this paper, the determinants for patient adherence to portal-supported rehabilitation treatments were explored from the perspective of patients. Where hardly any adherence issues were reported considering the offline elements of the treatment program, the usage of the portal applied within the treatment remained low. Although various determinants for adherence were identified, the most important barrier toward portal usage seemed to be uncertainty among patients about the fit of the portal within the treatment program and the importance of using it. From the determinants identified in the study, it seems that extrinsic motivation by care professionals plays an important role in countering these issues. The findings suggest that, to increase portal usage, the portal needs to be truly embedded in the overall treatment program, with a strong link to the activities scheduled in the offline sessions, and usage of the portal by patients should be actively stimulated by care professionals.

## Abbreviations

HBM: health belief model
ICT: information and communications technology
SA: supported accountability
SD: standard deviation
TAM: technology acceptance model
TPB: theory of planned behavior

## References

[1] Jansen-Kosterink S (2014). The added value of telemedicine services for physical rehabilitation.

[2] Wentzel J, van der Vaart R, Bohlmeijer ET, van Gemert-Pijnen JE (2016). Mixing online and face-to-face therapy: how to benefit from blended care in mental health care. JMIR Ment Health.

[3] Hamine S, Gerth-Guyette E, Faulx D, Green BB, Ginsburg AS (2015). Impact of mHealth chronic disease management on treatment adherence and patient outcomes: a systematic review. J Med Internet Res.

[4] Vance Wilson E, Lankton NK (2004). Modeling patients' acceptance of provider-delivered e-health. J Am Med Inform Assoc.

[5] Mohr DC, Cuijpers P, Lehman K (2011). Supportive accountability: a model for providing human support to enhance adherence to eHealth interventions. J Med Internet Res.

[6] Broens TH, Huis in 't Veld RM, Vollenbroek-Hutten MM, Hermens HJ, van Halteren AT, Nieuwenhuis LJ (2007). Determinants of successful telemedicine implementations: a literature study. J Telemed Telecare.

[7] Huis in 't Veld RM, Kosterink SM, Barbe T, Lindegård A, Marecek T, Vollenbroek-Hutten MM (2010). Relation between patient satisfaction, compliance and the clinical benefit of a teletreatment application for chronic pain. J Telemed Telecare.

[8] Vermeire E, Hearnshaw H, Van Royen P, Denekens J (2001). Patient adherence to treatment: three decades of research. A comprehensive review. J Clin Pharm Ther.

[9] Linn AJ, Vervloet M, van Dijk L, Smit EG, Van Weert JM (2011). Effects of eHealth interventions on medication adherence: a systematic review of the literature. J Med Internet Res.

[10] Viswanathan M, Golin C, Jones C, Ashok M, Blalock S, Wines R, Coker-Schwimmer EJ, Rosen DL, Sista P, Lohr KN (2012). Interventions to improve adherence to self-administered medications for chronic diseases in the United States: a systematic review. Ann Intern Med.

[11] Fishbein M, Ajzen I (1975). Belief, attitude, intention, and behavior: An introduction to theory and research.

[12] Ajzen I (1991). The theory of planned behavior. Organ Behav Hum Decis Process.

[13] Janz NK, Becker MH (1984). The Health Belief Model: a decade later. Health Educ Behav.

[14] Becker MH (1974). The Health Belief Model and sick role behavior. Health Educ Behav.

[15] Venkatesh V, Bala H (2008). Technology acceptance model 3 and a research agenda on interventions. Decis Sci.

[16] Holden RJ, Karsh BT (2010). The technology acceptance model: its past and its future in health care. J Biomed Inform.

[17] Braun V, Clarke V (2006). Using thematic analysis in psychology. Qual Res Psychol.

[18] Strauss A, Corbin J (1998). Basics of qualitative research: techniques and procedures for developing grounded theory.

[19] DiMatteo MR, Haskard KB, Williams SL (2007). Health beliefs, disease severity, and patient adherence: a meta-analysis. Med Care.

[20] Zolnierek KB, Dimatteo MR (2009). Physician communication and patient adherence to treatment: a meta-analysis. Med Care.

[21] Tabak M, Brusse-Keizer M, van der Valk P, Hermens H, Vollenbroek-Hutten M (2014). A telehealth program for self-management of COPD exacerbations and promotion of an active lifestyle: a pilot randomized controlled trial. Int J Chron Obstruct Pulmon Dis.

[22] Vollenbroek-Hutten M, Tabak M, Jansen-Kosterink S, Dekker M (2015). From telemedicine technology to telemedicine services. Proceedings of the 3rd 2015 Workshop on ICTs for improving Patients Rehabilitation Research Techniques (REHAB '15).

